# Nutritional Modulation of the Microbiota–Gut–Brain Axis in Cancer-Related Fatigue: A Narrative Review of Mechanisms, Biomarkers, and Interventions

**DOI:** 10.3390/nu18203371

**Published:** 2026-10-14

**Authors:** Jiaxin Luo, Xinyue Li, Bingxin Liu, Yinfang Yu, Xue Shen, Huiying Liu, Shimiao Hu, Qin Luo, Xinli Liang, Yanxia Xiong, Wenjun Liu

**Affiliations:** 1 School of Pharmacy, Jiangxi University of Chinese Medicine, Nanchang 330004, China; ljx161802@163.com (J.L.); lixinyue819@163.com (X.L.); liubx9@163.com (B.L.); yyf@crjz.com (Y.Y.); m18720061671@163.com (S.H.); luoqin2024@163.com (Q.L.); 2 State Key Laboratory for the Modernization of Classical and Famous Prescriptions of Chinese Medicine, Nanchang 330103, China; xiongyx@crjz.com; 3 Science and Technology Innovation Department, China Resource Jiangzhong Pharmaceutical Co., Ltd., Nanchang 330103, China; shenxue25@crjz.com (X.S.); lhy@crjz.com (H.L.)

**Keywords:** cancer-related fatigue, microbiota–gut–brain axis, nutrition, gut microbiota, dietary intervention, probiotics

## Abstract

**Background/Objectives:** Cancer-related fatigue (CRF) is one of the most prevalent and disabling symptoms among patients with cancer, but its underlying mechanisms and effective management strategies have not yet been fully elucidated. Increasing evidence suggests that the microbiota–gut–brain axis (MGBA) may contribute to the pathogenesis of CRF by integrating nutritional status, intestinal barrier integrity, microbial metabolites, immune responses, and host energy metabolism. However, direct causal evidence in human CRF remains limited, and several mechanistic insights are extrapolated from other disease models. This narrative review aims to synthesize evidence for nutritional MGBA–CRF regulatory mechanisms, focusing on microbiota-associated biomarkers and microbiome-targeted dietary interventions, and to distinguish directly observed in a CRF population from indirect hypothetical mechanisms. **Methods:** This review synthesizes current evidence on the nutritional regulatory mechanisms linking the MGBA to CRF, with particular attention to microbiota-associated biomarkers and dietary or microbiome-targeted interventions. **Results:** Microbiota dysbiosis, reduced production of short-chain fatty acids, disrupted tryptophan metabolism, and increased intestinal permeability may lead to systemic inflammation, neuroendocrine dysfunction, and impaired mitochondrial function, thereby exacerbating fatigue. Moreover, nutritional factors, dietary patterns, and microbiota-targeted interventions may help restore microbiota homeostasis and alleviate fatigue-related biological dysregulation. **Conclusions:** A deeper understanding of MGBA may help in developing personalized nutritional strategies for patients with cancer experiencing fatigue symptoms and support more effective management of CRF.

## 1. Introduction

### 1.1. Clinical Characteristics and Pathogenetic Hypotheses of CRF

Cancer-related fatigue (CRF) is a persistent, distressing, and multidimensional symptom commonly reported by patients with cancer during and after treatment [1]. Unlike ordinary fatigue, CRF cannot be fully alleviated by rest and is often accompanied by declined physical function, impaired quality of life, and reduced treatment tolerance [1].

CRF manifests across three interrelated yet clinically distinguishable domains, namely, physical, cognitive, and emotional fatigue [2,3]. Physical fatigue is characterized by decreased physical function, generalized weakness, and other manifestations [4,5]. It is usually assessed using the Piper Fatigue Scale or the Brief Fatigue Inventory (BFI) [6,7]. Cognitive fatigue includes symptoms such as reduced attention and slowed information-processing speed [8,9] and is typically assessed using tools such as the Functional Assessment of Cancer Therapy–Cognitive Scale (FACT-Cog) or objective neuropsychological tests [10]. Emotional fatigue manifests as emotional disturbances, anxiety, or depressive symptoms [11] and can usually be assessed using the Hospital Anxiety and Depression Scale (HADS) or the Profile of Mood States (POMS) [12,13]. These three dimensions often co-occur in patients with CRF and may share a common biological basis [14], such as systemic inflammation and neuroendocrine dysfunction [15]; however, there are also some distinct contributing factors, such as anemia and muscle atrophy, which can more directly lead to physical fatigue [16,17], whereas neuroinflammation in the prefrontal cortex may have a more significant impact on cognitive symptoms [18,19].

Although its pathogenesis involves multiple factors, increasing evidence suggests that CRF is closely associated with inflammatory activation, neuroendocrine dysfunction, metabolic disturbances, and changes in nutritional status [2,20,21] (Figure 1). Importantly, several of these mechanisms can directly cause fatigue without the involvement of the gut microbiota. Tumor-derived factors drive cachexia and skeletal muscle degradation [22,23], treatment-related myelosuppression causes anemia and tissue hypoxia [16], and reduced caloric intake limits adenosine triphosphate substrate availability [24]. These nonmicrobial pathways are well-established, independent contributors to CRF. Nevertheless, emerging evidence suggests that gut microbiota dysbiosis may interact with and amplify many of these processes by modulating systemic inflammation, energy harvest, and neuroendocrine function [25,26].

Notably, dietary intake serves as the primary substrate for microbial metabolism and directly shapes the composition and function of the gut microbial community [27]. Cancer-related anorexia, reduced food intake, treatment-induced gastrointestinal toxicity, and malnutrition may, therefore, disrupt gut microbiome homeostasis [28,29], impair intestinal barrier function [30], and alter microbial metabolite production, further exacerbating systemic inflammation and energy imbalance, ultimately contributing to the onset of fatigue [31].

### 1.2. Bidirectional Signaling Communication of MGBA

The microbiota–gut–brain axis (MGBA) closely links diet, gut microbiota, the immune system, and host metabolism through bidirectional signaling, providing a key framework for studying the perception of fatigue [32,33]. Specifically, the central nervous system (CNS) modulates intestinal peristalsis, immune responses, and the composition and activity of the gut microbiota through the hypothalamic–pituitary–adrenal (HPA) axis and autonomic nervous system [34]. Conversely, the gut microbiota and their metabolites regulate CNS functions via the enteric nervous system (ENS), vagus nerve, circulatory system, and immune system [35]. This bidirectional communication system maintains physiological homeostasis of the host, and its dysfunction contributes to the development of various neurological, psychiatric, and somatic symptoms [36] (Figure 2). Therefore, MGBA dysregulation may be a key factor in elucidating the mechanisms underlying the nutritional and metabolic disorders in CRF.

### 1.3. Multiple Roles of Gut Microbiota Dysbiosis in CRF

As an essential commensal community within the host, the gut microbiota is crucial in sustaining host health [37]. Gut dysbiosis is defined as “a state in which alterations in the microbial community disrupt symbiotic relationships and lead to or contribute to disease or dysfunction [38].” In patients with cancer, gut microbiota dysbiosis may be caused not only by tumor-associated inflammation, immune dysregulation, and intestinal barrier damage, but also by factors such as treatment-related gastrointestinal toxicity, antibiotic exposure, reduced dietary intake, and malnutrition [39,40,41]. These factors reduce the number of beneficial symbiotic bacteria, allowing opportunistic pathogens to proliferate, leading to disruption of the intestinal microecosystem [42].

The causal relationship between gut microbiota dysbiosis and CRF remains uncertain [25,26]. From a nutritional perspective, the same upstream factors that drive CRF (tumor burden, anticancer therapy, reduced food intake, and antibiotic exposure) can independently disrupt the gut microbiota [28,29,30,31,41]. Dysbiosis and fatigue may therefore co-occur as parallel consequences of these shared triggers, rather than one directly causing the other. At the same time, dysbiosis may act as a mediator that amplifies fatigue through the pathways reviewed below, whereas, fatigue and its drivers may themselves worsen dysbiosis. Consequently, malnutrition, inflammation, microbiota disturbance, and fatigue may reinforce one another, potentially forming a vicious cycle [35]. As most human studies are observational, the direction of causality cannot be definitively established [43,44,45,46]. Longitudinal studies with repeated microbiome sampling before, during, and after cancer treatment are therefore warranted to clarify these relationships.

While causality remains unproven, clinical evidence suggests a close association between gut dysbiosis and CRF [43,44,45]. In multiple malignancies such as breast cancer, head and neck cancer, and lung cancer, patients with CRF exhibit a marked decline in the abundance of short-chain fatty acid (SCFA)-producing beneficial bacteria along with a sharp increase in the abundance of pro-inflammatory opportunistic pathogens [43,44,45]. Such microbial structural changes are strongly correlated with the severity of fatigue [45,46]. This reflects the fact that gut microbiota dysbiosis should not be viewed merely as a microbial abnormality but rather as a nutrition-sensitive biological state that co-occurs with and potentially mediates the relationships among cancer treatment, dietary disorders, intestinal barrier dysfunction, systemic inflammation, and fatigue.

Fatigue perception involves the coordinated activity of multiple regions of the brain, including the nucleus tractus solitarius, hypothalamus, amygdala, prefrontal cortex, and locus coeruleus [47,48,49,50,51]. It has been proposed that changes in the gut microbiota may be relayed to the CNS via the MGBA [35], with signaling occurring primarily through three neural, immune, and endocrine/metabolic pathways [52]. The neural pathway may mediate the rapid transmission of microbial metabolite-related signals [35,53], the immune pathway may contribute to peripheral and central inflammation in the context of dysbiosis [54], and the endocrine/metabolic pathway may promote a chronic fatigue state through alterations in neurotransmitters and hormones [55,56,57]. Meanwhile, central fatigue generates descending feedback signals that further aggravate gut dysbiosis, potentially establishing a self-perpetuating vicious cycle [58].

Overall, while converging evidence supports the link between gut dysbiosis and CRF, the exact nature of this relationship is yet to be completely elucidated. Dysbiosis may act as a contributing factor, mediator, biomarker, or parallel epiphenomenon, or likely a combination of these, in the complex pathogenesis of CRF. Further longitudinal and interventional studies are therefore needed to unravel these relationships and identify therapeutic targets.

## 2. Materials and Methods

A comprehensive literature search was conducted in PubMed (n = 3976) and Web of Science (n = 5635) for articles published from 1997 to 26 January 2026, using two umbrella searches (CRF, MGBA) with 16 refinements (full search strings in Supplementary Table S1). Manual screening of the reference lists of eligible articles and of recent narrative and systematic reviews was also performed to identify any additional relevant records.

The search yielded 9611 records; after deduplication (n = 3690), 5921 records remained. Title/abstract screening led to the exclusion of 4178 off-topic records, leaving 1743 for full-text retrieval; 7 records were excluded due to the unavailability of full text articles. Of the 1736 full texts that were assessed, 1534 were excluded (fatigue not cancer-related: 1026; no nutrition/microbiota/inflammation content: 346; and did not address CRF mechanism: 162). A total of 202 studies were included. The selection process is summarized in Figure 3.

The 202 included studies differed in evidentiary weight and were organized into three functional tiers for reporting. The first tier, mechanistically relevant studies, comprised original clinical or preclinical investigations that directly quantified CRF-associated gut microbial taxa, microbial or host metabolites, inflammatory markers, or intestinal-barrier readouts in patients with cancer or in CRF animal models. The second tier, interventional trials, comprised prospective studies, predominantly randomized and pilot trials, testing a dietary, nutritional, probiotic, prebiotic, or synbiotic intervention with fatigue as a measured outcome. The third tier comprised the remaining studies, which provided contextual and mechanistic support and are cited inline; these include epidemiological and clinical background studies, mechanistic work in non-CRF models used for reasoned pathway extrapolation and flagged as indirect, and high-quality narrative and systematic reviews. They do not yield discrete CRF-specific biomarker or efficacy estimates and are therefore not tabulated, but each is individually cited at the proposition it supports.

Studies were eligible if they examined CRF in patients, survivors, or experimental models and addressed the gut microbiota, nutrition, microbial metabolites, inflammatory biomarkers, or microbiota-targeted interventions. Exclusion criteria were: no relation to CRF, absence of microbiome- or nutrition-related mechanisms, conference abstracts or letters with insufficient data, and unavailable full text.

As this was intended to be a narrative review, no formal meta-analysis or risk of bias assessment was conducted.

## 3. Nutrition-Related Mechanisms of MGBA Dysregulation in CRF

### 3.1. Neural System: Rapid Signal Transmission via the ENS and Vagus Nerve

The ENS and vagus nerve form a rapid, bidirectional gut–brain communication network [59] that is highly sensitive to nutritional status and microbial metabolism in CRF [36,60]. Nutrients, microbial metabolites, and enteroendocrine hormones modulate enteric neuronal excitability, vagal afferent firing, and sympathetic transmission; dietary disturbances and dysbiosis may thereby alter gut–brain neural signaling and elicit fatigue [36,60].

#### 3.1.1. Regulation of the Gut Microbiota–ENS–Brain Pathway

The ENS is referred to as the “second brain” [61], is a neuron–glial network in the gut wall that functions independently of, yet connects with, the CNS [62]. It relays intestinal signals to the CNS via enteric neurons and sympathetic ganglia [63,64] and senses luminal nutrients, microbial metabolites, and inflammatory signals; dietary components and their fermentation products influence intestinal neural activity, motility, secretion, and mucosal immunity [65]. The gut microbiota modulates this pathway via two mechanisms: (1) neurotransmitter secretion [66], with intestinal 5-HT neurons participating in gut–brain communication through the 5-HT-HTR7 pathway in the CNS and ENS [67], and (2) SCFAs and other metabolites activating receptors on ENS neurons to regulate excitability [68]. For example, butyrate activates GPR41/FFAR3 (high affinity) and GPR43/FFAR2 (lower affinity) [69,70], regulating ion-channel activity and signal propagation [71]. LPS activates TLR-associated pathways, inducing neuroinflammation and oxidative stress and impairing neuronal function [72]. Reduced food and fiber intake and treatment-related gastrointestinal toxicity decrease SCFA-producing bacteria, causing ion-channel dysfunction and increased LPS-driven neuroinflammation [73]. The microbiota–ENS–brain pathway thus links nutritional and microbial perturbations to CRF-related neurofunctional disorders [74]. However, while individual signaling components (5-HT-HTR7 [67], SCFA-GPR41/GPR43 [69,70], LPS-TLR [72]) are supported by preclinical evidence, direct demonstration of this complete pathway in CRF is lacking. Moreover, the proposed integration of these signals into CRF-related neurofunctional disorders remains hypothetical.

#### 3.1.2. Regulation of the Gut Microbiota–Vagus Nerve–Brain Pathway

The vagus nerve is the fastest, most direct gut–brain pathway, relaying visceral sensory signals in real time [59] and responding to both nutritional and microbial inputs [75]. Luminal nutrients stimulate enteroendocrine cells to release cholecystokinin, glucagon-like peptide-1, and 5-HT, which activate vagal afferents to regulate appetite, energy balance, and fatigue perception [76,77]. Microbial metabolites also signal via the vagus: SCFAs activate GPR41/GPR43 on vagal terminals and gut-associated epithelial cells, while 5-HT from enteroendocrine cells stimulates 5-HT3 receptors on vagal afferents [78]. These signals are integrated in the nucleus tractus solitarius and projected to fatigue-associated regions (hypothalamus, amygdala, prefrontal cortex) [79], potentially driving persistent fatigue [80]. This pathway is inferred from general MGBA research and from reduced SCFA-producing bacteria in patients with CRF [43,45,46], not from direct CRF vagal studies. In a neuropathic pain mouse model, butyrate analgesia depended on GPR41 expression (GPR41 knockout attenuated it) [81], supporting GPR41 in gut–brain microbial signaling, but its role in CRF is untested.

### 3.2. Immune System: Chronic Inflammation Along the Gut Microbiota–Immune System–Brain Axis

The immune system is a critical route for relaying microbial signals to the CNS [52,54]. Malnutrition and gastrointestinal toxicity cause dysbiosis, which compromises intestinal barrier integrity, accelerating systemic inflammation and directly modulating immune signaling [75,82]; these effects synergistically trigger peripheral and neuroinflammation, amplifying fatigue [83,84].

#### 3.2.1. Intestinal Barrier Disruption and Microbial Component Translocation

The intestinal barrier links nutrition, gut microbiota, and systemic immunity [85]. Tumor-and treatment-related dysbiosis reduces SCFA-producing bacteria, while insufficient dietary fiber limits butyrate and other SCFA synthesis, depriving epithelial cells of energy and reducing tight-junction protein expression, thereby compromising barrier integrity [86]. Leaky gut allows bacteria and their components to translocate into the blood, activating peripheral immune cells to release proinflammatory cytokines [64]. These factors cross vulnerable blood–brain barrier regions to infiltrate the brain parenchyma [87], activate cerebral endothelial cells, and recruit monocytes/macrophages into the CNS [88]; they also reach the nucleus tractus solitarius via the vagus [89], indirectly activating microglia and initiating central inflammatory cascades and neuroinflammation [90], which may drive CRF symptoms [91,92]. Therefore, nutritional intervention strategies that restore the supply of fermentable substrates, increase SCFA production, and maintain epithelial barrier integrity may help reduce immune activation and alleviate fatigue. Among these mechanisms, directly observed in a CRF population supports the clinical correlations between reduced SCFA-producing bacteria and the severity of fatigue [43,45,46] and the enrichment of pro-inflammatory taxa in patients with CRF [43]. However, the detailed molecular steps, including tight junction protein regulation by SCFAs [86], pathogen-derived hemolysin/protease degradation of tight junctions [86], and the specific cytokine–BBB–microglia cascade [87,88,90], are primarily extrapolated from general immunology and other disease models and have not been directly validated in CRF-specific contexts.

#### 3.2.2. Regulation of Immune Pathways by Microbial Components

Microbial components exert dual anti- and pro-inflammatory effects [93]. Anti-inflammatory: polysaccharide A from *Bacteroides fragilis* induces regulatory T cell differentiation via TLR2, increases IL-10, and suppresses central neuroinflammation [94]. Pro-inflammatory: LPS binds TLR4 and activates NF-κB, triggering IL-6 and TNF-α release [95]. In CRF, poor nutrition, low fiber, reduced SCFAs, and dysbiosis-related immune imbalance may exacerbate this inflammatory shift [28,30,44,82,85]. Clinically, circulating IL-6 and TNF-α correlate positively with fatigue severity in CRF [96,97], while anti-inflammatory SCFA producers decline and proinflammatory genera (*Family XIII AD3011*, *Erysipelatoclostridium*) increase [43]. *Family XIII AD3011* is abundant in colorectal-cancer-susceptible mice [98], and *Erysipelatoclostridium* correlates with LPS levels [99]; gene set enrichment links both genera to immune/inflammatory pathways [43]. Directly observed in a CRF population supports elevated IL-6 and TNF-α [100] and proinflammatory taxa enrichment with SCFA depletion [43]; the PSA–TLR2–Treg [94] and LPS–TLR4–NF-κB [95] mechanisms are extrapolated from preclinical/general immunology studies and require direct CRF confirmation.

### 3.3. Endocrine/Metabolic Pathway: Slow Signal Transmission Along the Gut Microbiota–Circulatory System–Brain Axis

The endocrine/metabolic pathway is the MGBA component most directly tied to nutrition [55,56]. The gut microbiota regulates neurotransmitter synthesis, tryptophan (TRP) metabolism, HPA axis activity, SCFA production, and host energy balance [36]. In patients with cancer, malnutrition, reduced intake, altered amino acid availability, and treatment-related metabolic stress may interact with dysbiosis to exacerbate fatigue [101]. Although biologically plausible, the specific interactions among nutritional deficiency, dysbiosis, and endocrine–metabolic dysfunction remain unvalidated in CRF; this section is a hypothetical synthesis.

#### 3.3.1. Neurotransmitter Regulation by the Gut Microbiota

CRF pathogenesis involves dysfunction of 5-HT, GABA, and dopamine (DA), linked to fatigue, reduced motivation, and mood disturbance [102,103,104]. These neurotransmitters and their precursors depend on dietary substrate supply and microbial metabolism [105]: dietary protein and TRP feed the 5-HT pathway, while dysbiosis redirects TRP toward immune-activating pathways [66,106]. Neurotransmitter alterations may reduce motivation, disturb sleep, impair cognition, and cause fatigue [20], with nutritional deficiency worsening precursor availability and microbial metabolite production [66]. Based on the literature on MGBA and neuropsychopharmacology, it can be inferred that 5-HT, GABA, and DA dysfunction is associated with CRF; however, studies directly measuring these neurotransmitter changes in patients with CRF are limited, and the hypothesis that nutritional deficiencies are linked to neurotransmitter dysfunction via microbial mediation remains purely speculative at this time.

The microbiota modulates neurotransmitter homeostasis via direct synthesis, precursor metabolism, and competitive degradation [107]: *Bifidobacterium infantis* raises plasma TRP to regulate 5-HT, and abnormal TRP metabolism is tied to CRF onset [108]; *Bacteroides*, *Parabacteroides*, and *Escherichia* produce GABA [109], which affects central function via the ENS and regulates depression/fatigue-related brain regions [107,109]. The gut microbiota participates in the metabolism of DA via specific enzymatic pathways for its modulation [107,110]. *Enterococcus* tyrosine decarboxylase converts L-DOPA to DA, and *Eggerthella* catechol dehydroxylase degrades DA [110,111], altering systemic dopaminergic tone and CNS function [110,111]. These mechanisms are unconfirmed in CRF-specific models or patients.

#### 3.3.2. Regulation of TRP Metabolism

TRP metabolism links nutrition, microbiome, immunity, and brain function [112] via the kynurenine (KYN), 5-HT, and indole pathways [113]. Kynurenic acid in the KYN pathway is neuroprotective [114], but inflammation shifts the pathway toward neurotoxic quinolinic acid, which activates NMDA receptors, causing excitotoxicity and neuronal death [115]. In the 5-HT pathway, TRP is converted to 5-HT by tryptophan hydroxylase 1 in enterochromaffin cells [116]. Inflammation-induced indoleamine 2,3-dioxygenase overactivation diverts TRP to the KYN pathway, reducing 5-HT substrate availability and central 5-HT levels while accumulating neurotoxic metabolites that drive neuroinflammation [117]. Reduced 5-HT plus quinolinic acid accumulation may underlie CRF-related central dysfunction. Clinically, breast cancer patients with CRF show decreased plasma TRP and increased KYN/TRP ratio, correlated with fatigue severity [46]; however, specific KYN enzyme dynamics are derived from general neurochemistry and other disease models, and their exact contribution to CRF is undetermined.

#### 3.3.3. Regulation of the HPA Axis by Gut Microbiota

Chronic stress, systemic inflammation, sleep disturbance, and nutritional deficiency cause HPA axis dysfunction in cancer patients, worsening fatigue and sleep [118]. Low energy/protein intake exacerbates metabolic stress, while dysbiosis-related inflammation alters glucocorticoid signaling and neuroendocrine feedback [118,119,120]. HPA dysfunction is clinically associated with CRF [121,122,123], but microbiota-mediated inflammation’s impact on HPA dysregulation in CRF is inferred from general MGBA literature and unvalidated in CRF-specific studies [124]. The gut microbiota activates the HPA axis via two routes [118,125]: the immune-mediated pathway, where dysbiosis-induced cytokines [125] and microbial components (LPS, peptidoglycan) [118] target the hypothalamus across the blood–brain barrier to trigger HPA activation [118]; and the neural-mediated pathway, where SCFAs modulate HPA-axis gene expression to mitigate stress [126] and activate the hypothalamic paraventricular nucleus via the vagus–NTS pathway [125]. Excessive HPA activation disrupts cortisol rhythms [127], linked to sleep disturbance and worsened morning fatigue in CRF [121,122,123]. Directly observed in a CRF population supports disrupted cortisol rhythmicity and its fatigue association [121,122,123]; microbial regulatory mechanisms (LPS/peptidoglycan HPA activation [118], SCFA/vagus–NTS HPA gene regulation [126]) come from preclinical/general MGBA studies.

#### 3.3.4. Regulation of SCFAs and Energy Metabolism

SCFAs (butyrate, acetate, propionate) link dietary fiber to host energy metabolism [128]. Butyrate is the energy substrate for colonic epithelial cells and is estimated to supply approximately 70% of their energy demand under physiological conditions [129]. In cancer, reduced fiber intake, dietary diversity, and treatment-related gastrointestinal toxicity decrease SCFA-producing bacteria, impairing epithelial energy supply and systemic energy homeostasis [130,131]. Butyrate influences mitochondrial function, skeletal muscle metabolism, thermogenesis, and energy expenditure via PGC-1α, UCP-1, and related regulators [132]. Clinically, head and neck cancer patients with low blood butyrate show more severe fatigue during and after treatment [133], confirming SCFAs’ role in CRF energy metabolism. Directly observed in a CRF population is the butyrate–fatigue association [133]; butyrate as a colonocyte energy substrate [129] and PGC-1α/UCP-1 regulation of mitochondria and thermogenesis [132] are inferred from general physiology and preclinical studies, with direct CRF relevance requiring further investigation.

### 3.4. Integrated Nutrition–Microbiota–Immune–Neuroendocrine Crosstalk

The three pathways of the MGBA work collectively to form an integrated network linking nutrition, the microbiome, the immune system, and the neuroendocrine system [36,52]. Diet shapes microbial metabolism, and metabolic byproducts regulate barrier function, immune responses, and neural signaling, collectively driving the perception of fatigue [64,117]. Therefore, CRF may represent a systemic disorder potentially driven by disruption of MGBA signaling linked to nutritional and microbiome imbalances [117]. The three pathways do not operate in isolation but converge through several shared mediators that couple neural, immune, and endocrine–metabolic signaling. SCFAs and TRP metabolites serve as central metabolic hubs [112,134], while ACh [135] and cortisol [119] act as bridging mediators coupling neural, immune, and endocrine branches.

Butyrate activates GPR41/GPR43 receptors expressed in the ENS and vagus nerve, rapidly transmitting intestinal signals [136,137], while also exerting anti-inflammatory effects by inhibiting histone deacetylases [138,139], whose overproduction is linked to neurological disorders such as Parkinson’s disease, schizophrenia, and depression [140]; furthermore, butyrate serves as an energy substrate that regulates systemic energy metabolism [128,129]. Whereas SCFAs primarily couple metabolic and neural signaling, TRP metabolism bridges endocrine and immune pathways. Under inflammatory conditions, TRP preferentially diverts to the KYN pathway, leading to the accumulation of neurotoxic quinolinic acid and subsequent neuronal damage [56,115], while reduced 5-HT substrate availability couples endocrine and neural dysfunction [56,112]. When metabolism shifts toward the 5-HT pathway, by contrast, 5-HT3 receptors on the vagus nerve are activated, providing an afferent signal that coordinates intestinal motility and immune function [67,78].

The cholinergic anti-inflammatory pathway provides a bridge between neural and immune branches: vagal efferent fibers secrete acetylcholine (ACh), which binds the α7 nicotinic ACh receptor (α7nAChR) expressed on immune cells to inhibit the synthesis of pro-inflammatory cytokines [135,141,142]. This brake is, however, fragile: gut dysbiosis impairs afferent vagal signaling and weakens the efferent anti-inflammatory output [143], whereas prolonged inflammatory stimuli blunt immune cell responsiveness to ACh [144,145,146]. Beyond neural–immune coupling, the neuroendocrine branch converges on the same inflammatory milieu through cortisol, the terminal HPA axis effector. Under physiological conditions, cortisol feeds back to suppress CRH and ACTH [144]; however, cytokine-driven HPA activation [119] and sustained pro-inflammatory stimuli [120,127] lead to sustained cortisol elevation that impairs glucocorticoid receptor sensitivity [120,127] and restrains neurogenesis and 5-HT/DA synthesis in the hippocampus [144,147]. Cortisol further feeds back onto the gut: it disrupts the intestinal barrier, curbs mucus secretion, and remodels intestinal peristaltic rhythm, thereby worsening gut dysbiosis [148,149,150]. Under physiological conditions, cortisol binding to mineralocorticoid receptors drives regulatory T cell differentiation and constrains inflammation [144]; however, under sustained dysbiotic inflammation, glucocorticoid receptor desensitization blunts this negative feedback, such that the pro-inflammatory phenotype is perpetuated rather than resolved [120,127]. The integrated cross-acting model indicates that CRF is not driven by any single pathway but by the cumulative effects of nutritional disruption, microbial imbalance, immune activation, and neuroendocrine dysregulation [20].

This integrated cross-acting model is a hypothetical framework that synthesizes evidence from multiple fields of research. Although the level of evidence supporting individual pathway components varies, the complete interactive network has not yet been directly validated as a comprehensive system in CRF. The proposed vicious cycle in the context of CRF therefore remains hypothetical and awaits direct empirical validation. A visual differentiation of the levels of evidence supporting each pathway segment using different line styles is depicted in Figure 4.

## 4. MGBA-Based Potential Biomarkers of CRF

### 4.1. Gut Microbiota Biomarkers

Numerous studies have verified that the initiation and progression of CRF are tightly associated with gut microbiota dysbiosis [43]. A decrease in the abundance of SCFA-producing bacteria and an increase in the enrichment of potentially pro-inflammatory bacterial communities may reflect a disruption in the microbial ecosystem in patients with CRF [45]. These microbial shifts are not only descriptive biomarkers of CRF, but may also indicate impaired dietary fiber utilization, reduced metabolic resilience, and altered host–microbe interactions [153] (Table 1).

### 4.2. Microbial Metabolites and Host Biomarkers

Among other potential biomarkers, seven major categories of MGBA-related candidate biomarkers for CRF are summarized in Table 2. These markers sequentially reflect gut microbiota dysbiosis, intestinal barrier impairment, systemic inflammatory cascades, central neurochemical dysregulation, and circulatory-metabolic abnormalities [44,91]. Collectively, they constitute an integrated biomarker system that connects gut dysbiosis with central fatigue through the MGBA, offering quantifiable evidence to clarify the multilevel pathological pathways underlying CRF [44].

In summary, systematic studies of patients with CRF and in vivo studies have demonstrated that dysbiosis of the gut microbiota, disturbances in microbial metabolites, abnormal inflammatory cytokines, and impaired intestinal integrity collectively regulate the onset and progression of CRF [91]. These biomarkers can provide valuable evidence for the diagnosis and mechanistic exploration of CRF.

## 5. Nutritional and Microbiota-Targeted Interventions for CRF

### 5.1. Nutritional Assessment Practices and Gaps in the Included Studies

Nutritional assessment in CRF research encompasses several complementary domains: dietary intake methods (food frequency questionnaires [FFQs], 24 h recalls, food records), diet quality indices (healthy eating index [HEI], Mediterranean Diet Adherence Screener [MEDAS], DII), anthropometric measures (body mass index [BMI], weight, waist circumference), body composition tools (bioelectrical impedance analysis [BIA], dual-energy X-ray absorptiometry [DXA], computed tomography [CT]-based skeletal muscle index), biochemical markers (albumin, prealbumin, fatty acid profiles), nutritional screening tools (Patient-Generated Subjective Global Assessment [PG-SGA], Malnutrition Screening Tool [MST], Nutritional Risk Screening [NRS] 2002), and malnutrition diagnostic frameworks (Global Leadership Initiative on Malnutrition [GLIM] criteria) [167,168,169,170].

Among the studies included in this review, dietary intake was assessed using FFQs [97], 24 h recalls [171,172], multiday food records [173,174], and Mediterranean diet-specific screeners (MEDAS [175], MedDietScore [176], 14-item MedDiet tool [173]). Diet quality was evaluated using the Healthy Eating Index (HEI)-2010 [97], Alternate Mediterranean Diet Score (aMED) [171], and DII [174]. Anthropometric measures (BMI, weight, waist circumference) were the most frequently used tools [97,174,175,176,177]. Body composition was assessed using BIA in two studies [175,178] and air-displacement plethysmography in one study [176]. Biochemical markers included serum fatty acids [179], albumin [178,180], and fructosamine [173].

Several important gaps were identified. Validated nutritional screening tools (PG-SGA, MST, NRS-2002) and GLIM diagnostic criteria were not used in any included study. DXA and CT-based skeletal muscle index, which are considered reference standards for body composition assessment in oncology [169], were absent, and longitudinal weight trajectory and standardized nutrition impact symptom assessment [181] were not reported. Three studies used fixed supplementation protocols without prospective dietary assessment [179,182,183], whereas one study relied on retrospective chart review [180]. These gaps limit the ability to distinguish the independent contribution of specific nutritional deficits to CRF and highlight the urgent need for a standardized nutritional assessment battery.

### 5.2. Dietary Intervention and Nutritional Modulation

Dietary intervention is a feasible and noninvasive strategy for modulating MGBA in patients with CRF [82]. Diets rich in dietary fiber, unsaturated fatty acids, and other nutritional patterns may help maintain the diversity of the gut microbiota, promote SCFA production, and reduce systemic inflammation [184]. Conversely, inadequate dietary intake and treatment-related malnutrition may exacerbate gut dysbiosis and fatigue [157].

Consistent with these mechanisms, a preclinical study provided direct experimental support. In mice, a high-fiber diet administered before and during 5-fluorouracil (5-FU) chemotherapy enriched propionate-producing taxa (*Bacteroidaceae* and *Akkermansiaceae*), increased cecal propionate concentrations, and attenuated neuroinflammation, reducing astrocyte reactivity in the hippocampal CA1 region and the midbrain. Notably, astrocyte density showed an inverse correlation with propionate levels and the abundance of these taxa [157]. As the study evaluated neuroinflammation rather than behavioral endpoints, it provides mechanistic proof of concept of how dietary fibers alleviate chemotherapy-related neuroinflammation, rather than directly demonstrating their ability to reduce fatigue. Against this mechanistic backdrop, Table 3 synthesizes the clinical evidence on dietary and nutritional interventions for CRF, summarizing study designs, findings, and effect sizes to guide practical dietary recommendations.

### 5.3. Probiotic, Prebiotic, and Synbiotic Interventions

Probiotics, prebiotics, and synbiotics are microbiome interventions for CRF. They alleviate inflammation and improve energy metabolism by restoring microbial balance, increasing SCFA levels, and improving intestinal barrier integrity [58,184]. Several clinical studies have shown that supplementation with probiotics or synbiotics can alleviate the symptoms of fatigue (Table 4). However, the safety profile of probiotics has not yet been conclusively established; research shows that probiotics may worsen outcomes in certain populations (e.g., patients receiving radiotherapy) [185], and the literature lacks systematic adverse-event reporting [186]; thus, putative benefits must be weighed against currently under-characterized risks.

To help readers grasp the overall evidence landscape of this review, the included studies were organized into the three functional tiers defined in Section 2: mechanistically relevant studies (Table 1 and Table 2) and interventional trials (Table 3 and Table 4) are tabulated, whereas contextual and supporting mechanistic studies are cited inline throughout the narrative. We have consolidated all clinical and preclinical studies cited in these tables into a single study-design overview matrix (Table 5). The matrix lists each cited study by its design type and sample size, organized by research theme; detailed methodological information for each reference is provided in the respective thematic tables (Table 1, Table 2, Table 3 and Table 4).

### 5.4. Future Prospects of Emerging Microbiota-Targeted Strategies and Multimodal Interventions

Fecal microbiota transplantation (FMT) represents an intervention capable of comprehensively restoring intestinal microbial homeostasis and has yielded robust therapeutic benefits against inflammatory bowel disease, recurrent *Clostridioides difficile* infection, and cancer immunotherapy [194,195]. Live biotherapeutic product (LBP) therapy employs defined, often genetically engineered bacterial strains to selectively modulate the gut microbiota and deliver specific therapeutic functions, offering targeted advantages for the management of intestinal and related disorders [196]. Both modalities have shown preliminary promise in mitigating gut and neurogenic inflammation in certain neurological conditions, including autism spectrum disorder [197], Alzheimer’s disease [198] and Parkinson’s disease [199], although the supporting evidence remains mixed and limited. Based on the MGBA mechanisms described above, FMT and LBP theoretically possess therapeutic potential for CRF-related interventions [200,201]. Nevertheless, no available clinical trials have directly assessed the anti-fatigue efficacy of FMT or LBP in patients with CRF. Small-scale proof-of-concept clinical trials are therefore warranted, with fatigue designated as the primary outcome measure to define their clinical utility for the treatment of CRF.

Additionally, the multifactorial nature of CRF onset and progression determines that single interventions may struggle to completely reverse its pathological progression [20,202]. Multimodal interventions that integrate microbiota-targeted approaches with behavioral modifications, such as physical activity, sleep hygiene, stress management, and pharmacotherapy, may produce synergistic therapeutic effects [202]. Existing studies have preliminarily demonstrated that a Mediterranean diet combined with exercise can significantly improve CRF in patients with breast cancer, thereby supporting the feasibility of multimodal strategies (Figure 5) [173,177]. Future studies should further explore comprehensive treatment approaches that combine gut microbiota restoration with nutritional and behavioral interventions.

## 6. Summary and Future Perspectives

### 6.1. Summary and Limitations of the Present Study

CRF is a multifactorial syndrome involving nutritional disturbances, dysbiosis, impaired barrier function, inflammation, neuroendocrine dysfunction, and energy metabolism disorders [20]. Using the MGBA as an integrative framework, this review specifically elucidates how dietary and nutritional issues reshape the gut microbiome, thereby impairing barrier function, activating immune inflammation, disrupting TRP and SCFA metabolism, interfering with neuroendocrine signaling, and ultimately inducing fatigue. This framework provides theoretical support for multitarget interventions for CRF and highlights the potential of dietary- and microbiome-modulation strategies as adjunctive therapies.

The current body of evidence has several limitations. First, the reported findings across studies are inconsistent. Differences in cancer type, stage of treatment, diet, sequencing methods, and assessment tools may explain the widespread inconsistencies in results across different studies. Second, direct CRF-specific mechanistic evidence remains scarce, and most mechanistic pathways are extrapolated from preclinical models or other disease contexts and have not been directly validated in patients with CRF. Third, the assessment of key variables is generally inadequate. Nutrition-related indicators, such as dietary intake, nutritional status, body composition, gastrointestinal symptoms, and oral nutritional support, are often not included in microbiome or biomarker analyses. There is also a lack of uniform standards for biomarkers from sample collection to clinical interpretation. Fourth, intervention studies are still in their early stages. Trials involving various diets and prebiotics, probiotics, and synbiotics exhibit significant heterogeneity in intervention protocols, dosages, study populations, and outcome measures. Furthermore, most studies do not concurrently measure the microbiome, metabolites, or inflammatory markers when assessing fatigue, which limits the reliability of the results and their clinical translation. Fifth, the microbiome analyses underlying the associations reported in Table 1 and Table 2 are subject to inherent methodological constraints. Most included studies report relative abundances derived from 16S rRNA or shotgun sequencing, which are compositional: the relative proportion of one taxon depends on the abundance of all other taxa, so an apparent decrease in a beneficial taxon may partly reflect the expansion of other taxa rather than a true absolute decline. Standard univariate tests (e.g., *t*-tests, Wilcoxon tests, Spearman correlations) do not account for this compositional structure and may yield spurious associations, whereas more robust approaches such as centered log-ratio transformation, ANCOM, or ALDEx2 are infrequently applied. In addition, microbiome studies typically test hundreds of taxa simultaneously, and inadequate correction for multiple testing (for example, false-discovery-rate adjustment) inflates the risk of false-positive associations. These analytical limitations should be borne in mind when interpreting the taxon-level and biomarker-level associations summarized in this review.

### 6.2. Future Research Directions

Large-scale, multicenter prospective cohort studies should be conducted in which fecal and blood samples from patients before, during, and after treatment should be analyzed. By integrating artificial intelligence and multiomics technologies, these studies should clarify the causal relationship between gut microbiota changes and CRF and identify reliable biomarker panels. Simultaneously, it is necessary to advance multimodal combined intervention strategies that integrate nutritional interventions, microbiome modulation, behavioral interventions, and pharmacological approaches. Standardized RCTs can provide evidence-based support for clinical treatment and facilitate the exploration of personalized, precision intervention protocols tailored to patients with different cancer types, treatment stages, and microbiome profiles.

## 7. Conclusions

Overall, the MGBA provides an integrated framework for understanding CRF, elucidating how nutritional disorders, dysbiosis, and metabolite imbalances may disrupt the intestinal barrier, trigger inflammatory cascades, and likely contribute to the onset and progression of CRF. Dietary interventions, probiotics, prebiotics, synbiotics, and other nutrition strategies targeting the microbiome show preliminary promise as adjunctive approaches for alleviating symptoms of fatigue and enhancing the quality of life of patients with cancer. Future studies could integrate nutritional assessments, microbiome analysis, metabolomics, inflammatory biomarkers, and standardized fatigue assessment measures to support the development of CRF-management strategies based on personalized nutrition.

## Figures and Tables

**Figure 1:**
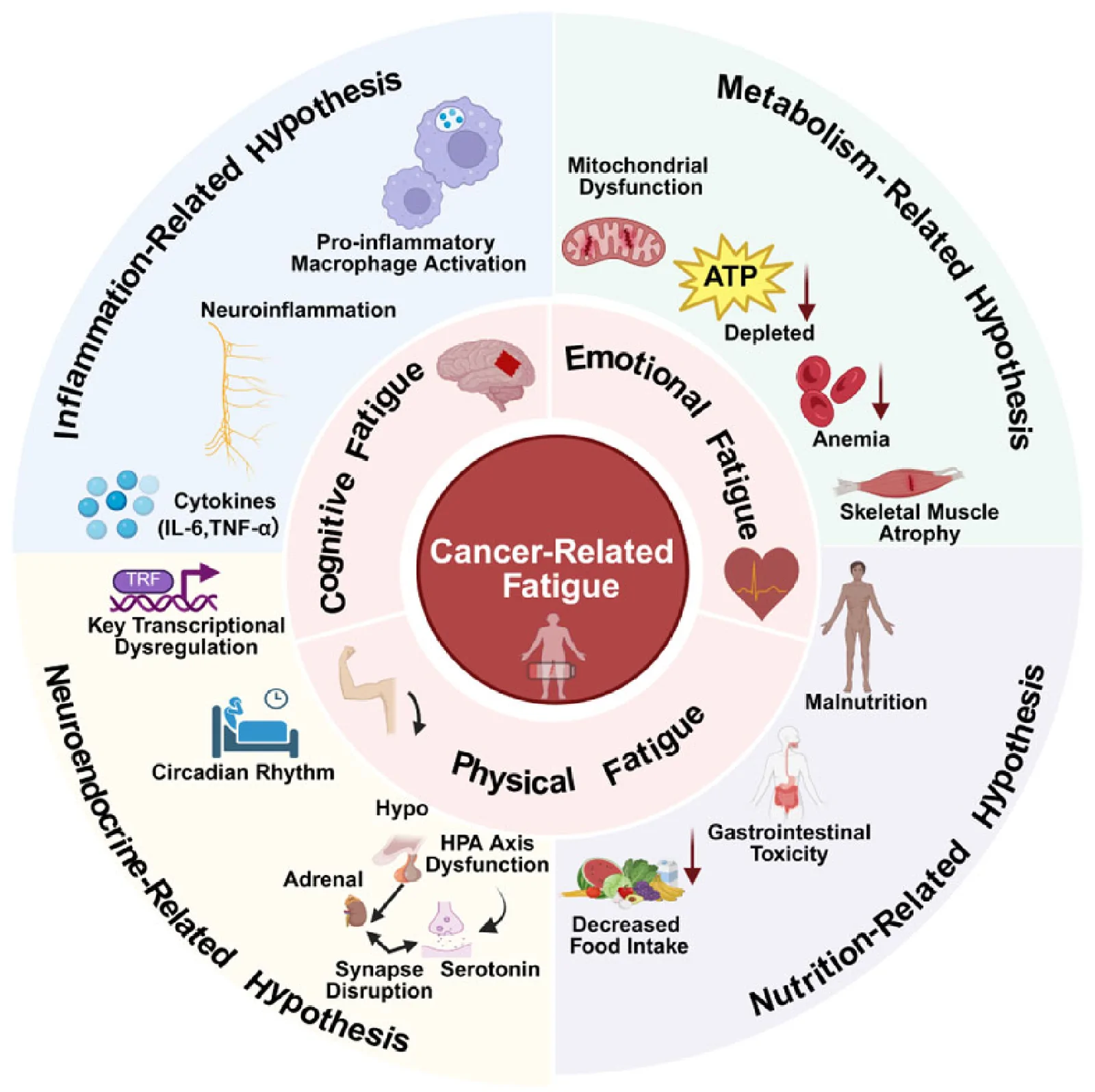
Clinical manifestations and core mechanistic hypotheses of CRF. CRF is mainly characterized by fatigue in physical, emotional, and cognitive domains [2,3]. Its pathogenesis may involve four interconnected pathways, namely inflammation, metabolic disorders, neuroendocrine dysregulation, and nutrition-related disturbances [2,20,21]. These mechanisms interact with each other and collectively form the complex regulatory network underlying CRF [20]. Red downward arrows indicate decreased levels of the corresponding factors (ATP, red blood cells, food intake). Created in BioRender. Luo, J. (2026) https://BioRender.com/z6lj808 (accessed on 10 October 2026).

**Figure 2:**
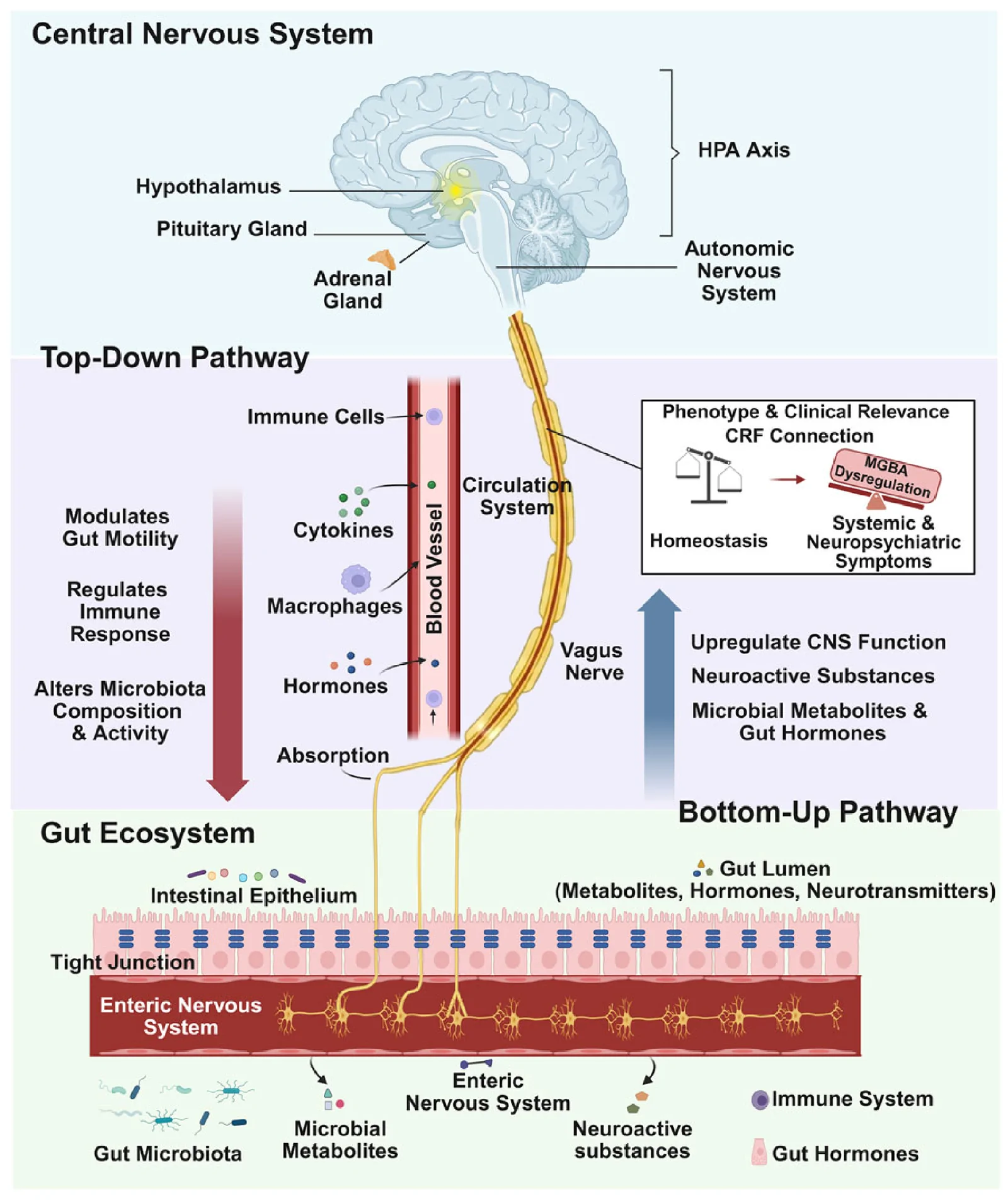
Bidirectional signal communication pattern of the MGBA. The MGBA mediates homeostatic regulation through bidirectional signaling between the CNS and the gut [32,33]. The CNS exerts descending regulation on the intestinal microenvironment via the HPA axis and autonomic nerves [34], the gut microbiota and their metabolites exert ascending regulation on central functions through neural, immune, and metabolic pathways [35]. Dysfunction of this axis disrupts bodily homeostasis and triggers neurological, psychiatric, and other related symptoms [36]. As an essential commensal community within the host, the gut microbiota is critical for sustaining host health [37]. Created in BioRender. Luo, J. (2026) https://BioRender.com/kjh6e6e (accessed on 10 October 2026).

**Figure 3:**
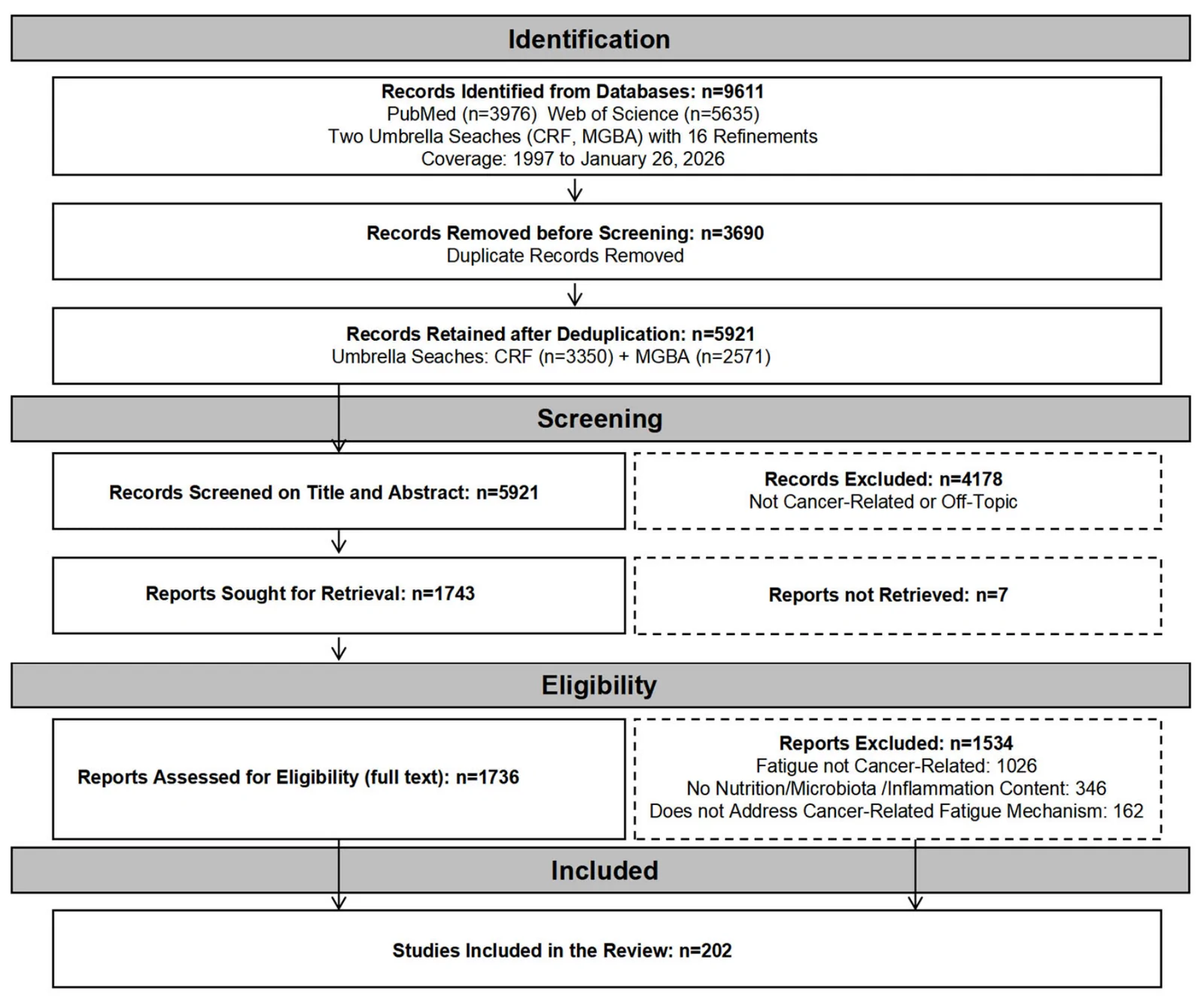
PRISMA-style flow diagram of the literature search and the study-selection process. PubMed and Web of Science were searched from 1997 to 26 January 2026, using two umbrella searches (CRF, MGBA) with 16 refinements. The reference lists of eligible articles were screened manually. The numbers at each stage represent the records retained or excluded; exclusion reasons are specified at the eligibility stage. Created using Microsoft PowerPoint.

**Figure 4:**
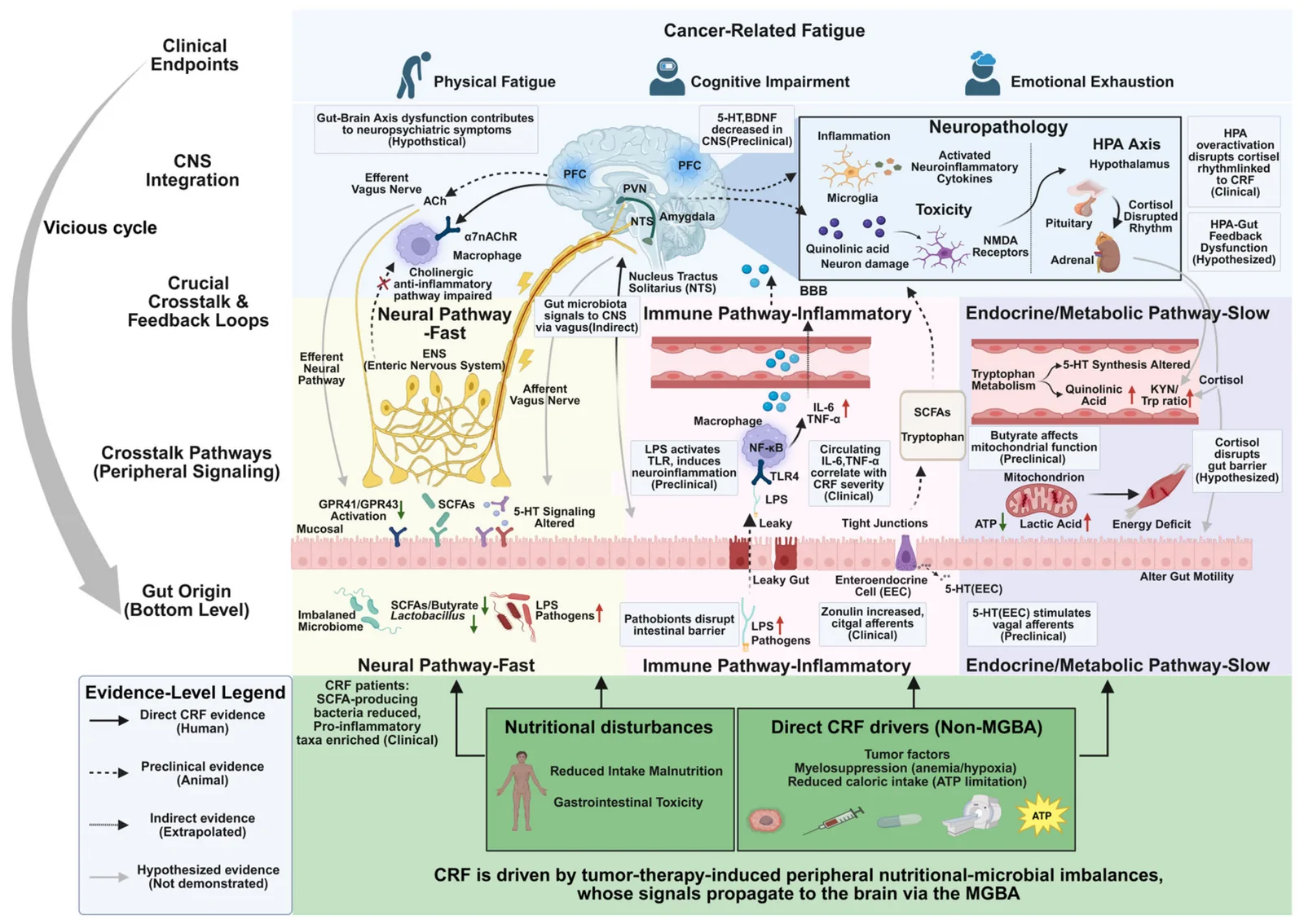
Integrated pathophysiology of CRF through the MGBA. Tumor burden, antitumor therapies, and nutritional disturbances drive gut dysbiosis [39,40,41], facilitating microbial metabolite translocation and peripheral immune activation [54]. Non-MGBA direct CRF drivers are shown for context: tumor-derived factors [22,23], myelosuppression [16], and reduced caloric intake [24]. CRF patients show reduced SCFA-producing bacteria and enriched pro-inflammatory taxa [43,44,45,46]. Dysbiosis propagates to the CNS via three branches—neural (fast), immune (inflammatory), and endocrine/metabolic (slow)—within the MGBA framework [52]. Neural: microbial signals are relayed via the ENS and vagus nerve [36,53]; 5-HT from enteroendocrine cells stimulates vagal afferents [78], and gut–brain dysfunction contributes to neuropsychiatric symptoms with decreased 5-HT/BDNF in the CNS [36,102]. Immune: compromised barrier integrity [86] and increased zonulin [151] facilitate LPS translocation, activating the NF-κB/TLR4 cascade and pro-inflammatory cytokines (IL-6, TNF-α) [95] that correlate with CRF severity [96] and cross the BBB to activate microglia [90,95]. Endocrine/metabolic: disturbed TRP homeostasis [56,112] and reduced SCFAs [128,134] impair butyrate-mediated mitochondrial function [132]; HPA overactivation disrupts cortisol rhythmicity [121,122,123,127], causing neurotoxicity [56], mitochondrial dysfunction [132,152], and energy deficiency [128,129]. Cascades converge on physical fatigue, cognitive impairment, and emotional exhaustion. Branches are interconnected by the cholinergic anti-inflammatory pathway [135], HPA negative feedback [144], and cortisol-mediated barrier disruption [148,149,150]; HPA–gut feedback dysfunction sustains the vicious cycle, whereby CNS dysfunction worsens dysbiosis via the autonomic nervous system and HPA axis [34,58]. Arrow styles: solid = direct CRF (humans); dashed = preclinical; dotted = indirect (extrapolated); gray = hypothesized. Inline boxes specify evidence type and references. Red crosses denote inhibition; Green downward arrows denote decreased expression, and red upward arrows denote increased expression. Created in BioRender. Luo, J. (2026) https://BioRender.com/18mjoxd (accessed on 10 October 2026).

**Figure 5:**
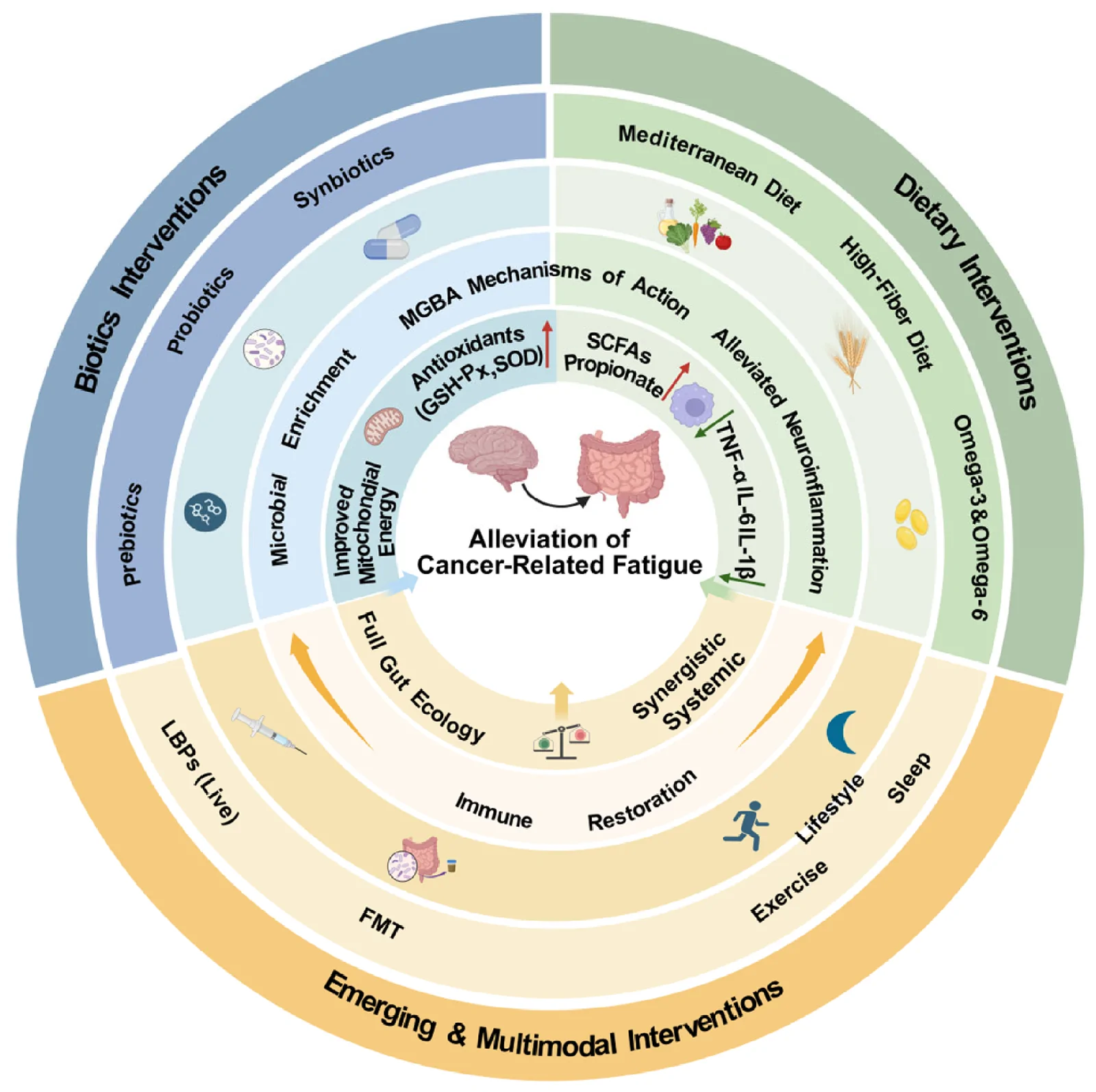
MGBA-targeted intervention strategies for CRF. The concentric circle model illustrates three levels of intervention strategies targeting the MGBA to alleviate CRF. Dietary intervention works by increasing SCFAs and reducing neuroinflammation [82,157]. Biological agent intervention acts by enriching beneficial bacteria, enhancing antioxidant enzyme activity, and improving mitochondrial energy metabolism [58,184]; emerging and multimodal interventions combined with lifestyle intervention amplify therapeutic effects by reconstructing the gut microecology, restoring immune function, and achieving synergistic regulation [194,202]. The three levels of strategies jointly alleviate CRF [58]. Green downward arrows denote decreased expression, and red upward arrows denote increased expression. Created in BioRender. Luo, J. (2026) https://BioRender.com/uqdv1q3 (accessed on 10 October 2026).

**Table 1:** CRF-related gut microbial biomarkers.

Taxonomic Rank	Taxon	Assoc. with CRF	Cancer Type	Study Type	Study Population	Analysis Method	Ref
Phylum	*Actinobacteriota*	↑	Colorectal (mouse)	Preclinical	C57BL/6 mice (AOM/DSS ± 5-FU)	16S rRNA; grip/running endurance; FMT	[154]
	*Proteobacteria*	↑	Breast (chemo)	Prospective cohort	100 pts (58 mod/severe, 42 mild)	16S rDNA; logistic regression (AUC = 0.82)	[44]
	*Proteobacteria*	↑	Colorectal (mouse)	Preclinical	C57BL/6 mice	16S rRNA; FMT	[154]
	*Verrucomicrobia*	↑	Colorectal (mouse)	Preclinical	C57BL/6 mice	16S rRNA; FMT	[154]
	*Bacteroidota*	↓	Breast (diet, cross-sectional)	Cross-sectional	342 pts (149 CRF; 64 stool 16S)	16S rRNA; differential abundance (FDR-corrected)	[46]
		↓	Colorectal (mouse)	Preclinical	C57BL/6 mice	16S rRNA; FMT	[154]
	*Firmicutes*	↓	Breast (chemo)	Prospective cohort	100 pts	16S rDNA	[44]
		↓	Colorectal (mouse)	Preclinical	C57BL/6 mice	16S rRNA; FMT	[154]
Class	*Actinobacteria*	↑	Colorectal (mouse)	Preclinical	C57BL/6 mice	16S rRNA; FMT	[154]
	*Gammaproteobacteria*	↑	Breast (chemo)	Prospective cohort	100 pts	16S rDNA; LEfSe	[44]
Order	*Enterobacteriales*	↑	Breast (chemo)	Prospective cohort	100 pts	16S rDNA; LEfSe	[44]
Family	*Enterobacteriaceae*	↑	Lung (adv, 1st-line chemo)	Pilot (mild/severe)	20 pts (10/10)	16S rRNA; LEfSe; Random Forest; KEGG	[45]
	*Ruminococcaceae*	↓	Breast (chemo)	Prospective cohort	100 pts	16S rDNA; LEfSe	[44]
		↓	Head & neck	Pilot (prospective)	13 HNC (high/low fatigue)	16S rRNA; DNA methyl.; LEfSe; GSEA	[43]
		↓	Lung (adv, 1st-line chemo)	Pilot (mild/severe)	20 pts	16S rRNA; LEfSe	[45]
	*Lachnospiraceae*	↓	Lung (adv, 1st-line chemo)	Pilot (mild/severe)	20 pts	16S rRNA; LEfSe	[45]
Genus	*Escherichia-Shigella*	↑	Breast (diet, cross-sectional)	Cross-sectional	342 pts (64 stool 16S)	16S rRNA; dominant only in CRF (descriptive)	[46]
		↑	Breast (mouse)	Preclinical	BC post-chemo CRF mice	16S rRNA; correlation	[100]
		↑	Lung (adv, 1st-line chemo)	Pilot	20 pts	16S rRNA; LEfSe	[45]
	*Bifidobacterium*	↑	Breast (diet, cross-sectional)	Cross-sectional	342 pts (64 stool 16S)	16S rRNA; dominant only in CRF (descriptive)	[46]
	*Burkholderia-Caballeronia-Paraburkholderia*	↑	Breast (mouse)	Preclinical	BC post-chemo CRF mice	16S rRNA; correlation	[100]
	*Streptococcus*	↑	Breast (mouse)	Preclinical	BC post-chemo CRF mice	16S rRNA; correlation	[100]
		↑	Rectal (post-CRT)	Cross-sectional pilot	50 pts (35/15)	16S rRNA; QIIME2; LEfSe	[155]
	*Cosenzaea*	↑	Mixed (adv metastatic)	Cross-sectional	88 pts	16S rRNA	[156]
	*Family XIII AD3011*	↑	Head & neck	Pilot	13 HNC	16S rRNA; LEfSe	[43]
	*Erysipelatoclostridium*	↑	Head & neck	Pilot	13 HNC	16S rRNA; LEfSe	[43]
	*Veillonella*	↑	Breast (chemo)	Prospective cohort	100 pts	16S rDNA; logistic; LEfSe	[44]
	*Megasphaera*	↑	Breast (chemo)	Prospective cohort	100 pts	16S rDNA; LEfSe	[44]
	*Eubacterium*	↑	Rectal (post-CRT)	Cross-sectional pilot	50 pts	16S rRNA; LEfSe	[155]
	*Adlercreutzia*	↑	Rectal (post-CRT)	Cross-sectional pilot	50 pts	16S rRNA; LEfSe	[155]
	*Actinomyces*	↑	Rectal (post-CRT)	Cross-sectional pilot	50 pts	16S rRNA; LEfSe	[155]
	*Bacteroides*	↓	Breast (diet, cross-sectional)	Cross-sectional	342 pts (64 stool 16S)	16S rRNA; dominant only in NCRF (descriptive)	[46]
	*Prevotella*	↓	Breast (diet, cross-sectional)	Cross-sectional	342 pts (64 stool 16S)	16S rRNA; dominant only in NCRF (descriptive)	[46]
	*Sutterella*	↓	Breast (diet, cross-sectional)	Cross-sectional	342 pts (64 stool 16S)	16S rRNA; differential abundance (FDR-corrected)	[46]
	*Lachnospiraceae_UCG-010* (clade)	↓	Breast (diet, cross-sectional)	Cross-sectional	342 pts (64 stool 16S)	16S rRNA; differential abundance (FDR-corrected)	[46]
	*Eubacterium_oxidoreducens_group* (clade)	↓	Breast (diet, cross-sectional)	Cross-sectional	342 pts (64 stool 16S)	16S rRNA; differential abundance (FDR-corrected)	[46]
	*Candidatus Arthromitus*	↓	Breast (mouse)	Preclinical	BC post-chemo CRF mice	16S rRNA; correlation	[100]
	*Lactobacillus*	↓	Breast (mouse)	Preclinical	BC post-chemo CRF mice	16S rRNA; correlation	[100]
	*Clostridia_UCG-014* (unclassified family)	↓	Breast (mouse)	Preclinical	BC post-chemo CRF mice	16S rRNA; correlation	[100]
	*Subdoligranulum*	↓	Head & neck	Pilot	13 HNC	16S rRNA; LEfSe	[43]
	*Faecalibacterium*	↓	Head & neck	Pilot	13 HNC	16S rRNA; LEfSe	[43]
	*Agathobacter*	↓	Head & neck	Pilot	13 HNC	16S rRNA; LEfSe	[43]
	*Bifidobacterium*	↓	Head & neck	Pilot	13 HNC	16S rRNA; LEfSe	[43]
	*Phascolarctobacterium*	↓	Breast (chemo)	Prospective cohort	100 pts	16S rDNA; logistic	[44]
	*Blautia*	↓	Breast (chemo)	Prospective cohort	100 pts	16S rDNA; sub-study 16S	[44]
	*Lachnospiraceae-UCG-008*	↓	Lung (adv, 1st-line chemo)	Pilot	20 pts	16S rRNA; LEfSe	[45]

Notes: ↑, positive association with CRF; ↓, negative association with CRF.

**Table 2:** Candidate MGBA-related biomarkers of CRF.

Biomarker Domain	Representative Biomarkers	Typical Change in CRF	Clinical or Biological Significance	MGBA Linkage	Ref
Microbial metabolites	Butyrate, Propionate	Decreased	Decreased SCFAs induce barrier dysfunction and immune and neuroinflammatory activation	Strong: Gut microbial fermentation products reflecting compositional and functional shifts in the microbiota	[133,157]
TRP metabolism	TRP KYN/TRP ratio	Decreased Increased	Abnormal TRP metabolism connects gut dysbiosis with neuroimmune dysfunction and central fatigue	Strong: Microbial TRP metabolism directly influences CNS serotonin and kynurenine signaling	[46]
Endotoxin-related markers	LPS	Increased	Higher microbial products reflect impaired gut barrier and systemic inflammatory signals	Strong: Microbial product translocation across the compromised gut barrier; a core MGBA mechanism	[96]
Intestinal barrier markers	Zonulin Citrulline	Increased Decreased	Abnormal expression induces intestinal barrier dysfunction, disrupting intestinal and blood–brain barrier integrity and triggering peripheral and central inflammation	Strong: Gut barrier permeability markers; a core MGBA component	[151,158,159,160]
Inflammatory mediators	IL-6, TNF-α, IL-1β, CRP	Increased	Elevated inflammatory signaling is consistently implicated in the pathophysiology of CRF	Moderate: Broad systemic markers with wide natural variation; MGBA attribution requires mediation or multivariable studies	[100,154,157,161]
Energy and oxidative stress markers	Guanidinoacetic acid Mitochondrial DNA GSH-Px, SOD, NAD-MDH	Increased Decreased Decreased	Disturbed bioenergetics and redox homeostasis may contribute to low vitality and exercise intolerance	Moderate: Mitochondrial dysfunction may be secondary to MGBA-mediated inflammation; not primarily MGBA-linked	[152,162,163]
Neurotransmitter-related markers	5-HT, Brain-derived neurotrophic factor (BDNF)	Decreased	Neurochemical disturbance may underlie cognitive, affective, and motivational dimensions of CRF; direct BDNF measurement in CRF is limited and mainly preclinical	Moderate: May be modulated by MGBA (e.g., microbial SCFA and TRP pathways) but influenced by multiple pathways; MGBA mediation not demonstrated	[44,46]
Hematological markers	Hemoglobin	Decreased	Anemia (decreased hemoglobin) is a well-established contributor to CRF; higher hemoglobin levels are associated with significantly fewer instances of patient-reported fatigue	Low MGBA linkage: Hematological/oxygen-carrying measure; not an MGBA marker; included as confounder and differential diagnosis	[164,165]
Endothelial dysfunction markers	von Willebrand factor, soluble thrombomodulin	Increased	Endothelial dysfunction may contribute to microcirculatory impairment and hypoxia, linked to CRF severity	Low MGBA linkage: Vascular markers not directly related to the MGBA pathway; included as systemic correlates	[166]

**Table 3:** Dietary and nutritional interventions for CRF.

Cancer Type	n	Design	Nutritional Strategy	Nutrition Assessment (Method & Result)	Nutrition–CRF Link	CRF Measure & Effect	Ref
Breast cancer survivors (stage I–IIIA)	633	Observational cohort (HEAL)	ω-3 vs. ω-6 fatty acid intake	WHI FFQ + HEI-2010 + BMI; higher ω-6:ω-3 ratio increased CRP (*p* = 0.01)	Higher ω-3 relative to ω-6 may lower inflammation (CRP) and reduce physical fatigue	Piper Fatigue Scale + SF-36; fatigue increased with CRP tertile (*p* ≤ 0.01); high CRP = 1.8× fatigue odds (*p* < 0.05)	[97]
Breast cancer survivors (4–36 months post-treatment)	108	Multicenter RCT (phase II)	ω-3 vs. ω-6 PUFA supplementation (6 g/d, 6 weeks)	Serum fatty acid/lipid biomarkers (compliance); O6-PUFA lowered TNF-α pathway inflammatory markers vs. O3	O6-PUFA lowered TNF-α pathway inflammation, paralleling its larger CRF reduction	Symptom inventory (primary); SI-CRF decreased by 2.51 (O6) vs. 0.93 (O3) pts (ES −0.86, *p* < 0.01; d = 0.73)	[179]
Advanced NSCLC (stage IIIb–IV)	92	RCT (first-line chemo)	EPA-enriched oral nutritional supplement (ONS-EPA)	Intake records + BIA + albumin; ONS-EPA increased energy/protein intake (*p* < 0.001), LBM + 1.6 vs. −2.0 kg (*p* = 0.01)	Improved nutritional status (energy/protein, LBM gain, anti-cachexia) accompanied reduced fatigue/anorexia	EORTC QLQ-C30; EORTC QLQ-C30; fatigue decreased in the ONS-EPA arm (*p* ≤ 0.05); LBM gain *p* = 0.01	[178]
Stage I–III cancer (91% breast), on chemo	33	Pilot RCT	Mediterranean diet intervention	14-item MedDiet tool + 3-day records; >70% increased adherence (score +2.7 vs. +0.7, *p* < 0.05); fructosamine improved (ES −0.55)	Higher adherence lowered fatigue; MedDiet improved T-cell mitochondrial function (a fatigue correlate)	FACIT-F + Brief Fatigue Inventory; fatigue change ES 0.31/0.25 (CIs cross 0, NS); low-adherence subgroup ES 0.67/0.48	[173]
Patients with cancer (NHANES 2017–2020)	707	Cross-sectional	Mediterranean diet adherence (aMED score)	aMED from NHANES 24 h recall; higher aMED lowered fatigue (β −0.121 to −0.074, *p* ≤ 0.007; dose dependent)	Higher MedDiet adherence directly, dose-dependently lowered fatigue	Patient-reported fatigue (β coefficients above)	[171]
Breast cancer survivors (DEDiCa trial)	227	Single-arm 12-mo lifestyle program	Low-GI Mediterranean diet + exercise + vitamin D	7-day food records + anthropometrics; MedDiet adherence + 15%/20%; GI/DII decreased, CRP −12%, WC −4 cm, weight −1.9 kg	Improved diet quality (higher adherence, lower GI/DII) and weight loss accompanied fatigue reduction	EORTC QLQ-C30 fatigue subscale decreased vs. baseline (*p* < 0.001); single-arm; no control group	[174]
Prostate cancer on ADT	23	Pilot RCT	Mediterranean-style diet	MEDAS (14-item) + BIA + BMI; 81% adherence; body mass −2.97 kg, lean mass −1.50 kg (*p* ≤ 0.036), IL-8 −0.18 ng/mL (*p* = 0.029)	Reduced body/lean mass and IL-8 paralleled CRF improvement (FACIT-F +4.8/+7.2)	FACIT-F. MedDiet increased CRF vs. usual care: +4.8 (*p* = 0.05) at 8 weeks, +7.2 (*p* = 0.005) at 12 weeks	[175]
Breast cancer survivors (real-world program)	96	Nonrandomized, single-arm (3 months)	Therapeutic exercise + Mediterranean-diet education	Modified MedDiet questionnaire + anthropometrics; adherence based (no detailed nutrient data reported)	Exercise + MedDiet education lowered CRF (program level; no separate nutrition–CRF estimate)	CRF decreased pre to post (*p* = 0.005); QoL & limb function improved; single-arm, non-RCT	[177]
Breast cancer (on chemo)	49	Double-blind RCT (3 months)	Melatonin (1 mg/day) + Mediterranean diet (both arms)	MedDietScore + Bod Pod body composition; both arms showed increased adherence & lowered BMI; baseline CRF correlated with BMI (β −0.882), MedDietScore correlated with FACIT-F (β 0.389)	Higher MedDiet adherence and lower BMI/body fat lowered CRF; melatonin arm showed improved CRF vs. baseline	FACIT-F. Melatonin arm showed improved CRF vs. that at baseline (*p* = 0.003); no between-group difference (both on Mediterranean diet)	[176]

Notes: Effect sizes (Cohen’s d/Hedges’ g) and 95% confidence intervals (CIs) are given where reported; in small pilot/underpowered trials, statistical significance does not necessarily indicate a clinically meaningful improvement in CRF.

**Table 4:** Probiotic and prebiotic interventions for CRF.

Cancer Type	n	Design	Nutritional Strategy	Nutrition Assessment (Method & Result)	Nutrition–CRF Link	CRF Measure & Effect	Ref
Colorectal cancer (stage II–III), post-treatment survivors	60	Double-blind, randomized, placebo-controlled trial; 12 weeks	Probiotic *Lactobacillus rhamnosus R0011 + Lactobacillus acidophilus R0052*	Fixed supplement (2 capsules/day, 12 weeks); no dietary record. Exploratory: bowel-symptom & QoL questionnaires	Probiotic may restore microbial balance and strengthen intestinal barrier, lowering systemic/neuroinflammation and fatigue	FACT-Fatigue subscale (higher = less fatigue): probiotic arm 43.00 (IQR 36.50–45.50) to 44.50 (38.50–49.00) at 12 weeks (within-group *p* = 0.02); between-group cancer-related FACT change *p* = 0.04. Small arm (n = 28 probiotic/32 placebo)	[182]
Colorectal cancer, on chemotherapy	140	Double-blind RCT; probiotic 4 weeks + omega-3 8 weeks	Microbial cell preparation (probiotic, 30 billion CFU/sachet × 2/day) + omega-3 (2 g/day)	Fixed supplementation; inflammatory markers measured: serum IL-6 decreased in intervention (*p* = 0.002); placebo IL-6 and TNF-α increased (*p* = 0.004, *p* = 0.048)	Lower systemic inflammation (IL-6) is implicated in CRF pathophysiology	EORTC QLQ-C30 fatigue subscale (higher = worse): intervention 23.70 ± 2.80 to 11.97 ± 1.80 at 8 weeks (*p* < 0.05); placebo 14.23 ± 2.90 to 31.10 ± 3.00 at 8 weeks (*p* < 0.05, worsened). Note baseline imbalance (intervention started higher)	[183]
Breast cancer, on chemotherapy	67	Randomized placebo-controlled double-blind; 8 weeks	Synbiotics: 12 probiotic strains + fructooligosaccharide prebiotic	Oral synbiotics twice daily, 8 weeks; 24 h dietary recall at 0/4/8 weeks (confounders adjusted: protein, fat, fiber, Ca, vitamin C)	Synbiotics may rebalance the gut microbiota and increase SCFA levels, attenuating chemo-related fatigue via barrier/anti-inflammatory effects	Cancer Fatigue Scale (higher = worse): synbiotics arm fell at week 4 (*p* < 0.001) and week 8 (*p* < 0.001) vs. baseline; placebo increased (*p* = 0.032 at 8 weeks); between-group *p* < 0.001 at 8 weeks (adjusted)	[172]
Mixed cancers (breast, CNS, colorectal, etc.); mostly advanced	54	Multicenter retrospective study	Integrative: multistrain probiotics + vitamins C/D + medicinal-mushroom blend (U-CARE)	Retrospective chart review; no prospective dietary/nutrition assessment	Probiotics and mushroom blend may modulate microbiome and inflammation	44.4% (24/54) improved in both fatigue and QoL (clinical/self-report; Brief Fatigue Inventory cited but authors note no dedicated questionnaire was used)	[180]
Esophageal carcinoma, post-radiotherapy	80	Randomized controlled trial; HEN + probiotics vs. routine enteral nutrition	HEN + Bifid triple viable probiotic capsules	Nutritional indices (albumin, Hb, prealbumin, transferrin, TSF, AMC) and gut microbiota (*Bifidobacterium*, *Lactobacillus*, etc.) measured; intervention improved all vs. control (*p* < 0.05)	Better nutrition and microbiota recovery may reduce CRF	Fewer fatigued patients in intervention vs. control (*p* < 0.05); full text reports fatigue-free proportion ~30 percentage points higher	[187]

Notes: Several trials are small or underpowered pilots (e.g., n = 60 [182] and n = 67 [172]). In such studies, statistical significance does not necessarily reflect a clinically meaningful improvement in CRF. We have therefore reported effect sizes/direction and avoid the unqualified phrase “significant improvement.” Fatigue instruments differ in score direction: FACT—Fatigue higher = less fatigue; EORTC QLQ-C30 fatigue subscale and Cancer Fatigue Scale—higher = worse.

**Table 5:** Overview of study designs cited in Table 1, Table 2, Table 3 and Table 4, organized by research theme.

Study Design	Microbiome Composition (Table 1)	Host Biomarker (Table 2)	Dietary Intervention (Table 3)	Microbiota-Targeted Intervention (Table 4)
RCT	—	—	[162] (n = 108); [161] (n = 92); [187] (n = 80); [156] (n = 67); [165] (n = 60); [159] (n = 49)	[166] (n = 140); [187] (n = 80); [156] (n = 67)
Nonrandomized/single-arm intervention	—	—	[160] (n = 96)	—
Prospective cohort	[44] (n = 100)	[78] (n = NR); [116] (n = NR); [188] (n = NR)	[79] (n = 633)	—
Cross-sectional/case–control	[46] (n = 342); [147] (n = 88); [189] (n = 50); [45] (n = 20); [43] (n = 13)	[136] (n = NR)	[155] (n = 707)	—
Retrospective/uncontrolled	—	—	[151] (n = 227)	[163] (n = 54)
Systematic review	—	[190]	—	—
Other clinical	—	[137,148,149,150,191]	—	—
Preclinical	[82,192]	[134,144,150,192,193]	[144]	[134]

Notes: A: Studies cited in Table 1, Table 2, Table 3 and Table 4 are listed by study design and research theme. Sample sizes (n) are given where reported; “NR” indicates the sample size was not reported in the original study, and “—” indicates that the corresponding design category was not represented in that thematic table. Preclinical studies are listed separately as mechanistic references. This matrix is descriptive and does not constitute a formal risk-of-bias or quality assessment; readers should consult the individual thematic tables and primary publications for detailed methodological appraisal.

## Data Availability

The original contributions presented in this study are included in the article/Supplementary Materials. Further inquiries can be directed to the corresponding authors.

## Supplementary Materials

The following supporting information can be downloaded at https://www.mdpi.com/article/10.3390/nu18203371/s1, Table S1: Full search strings used in PubMed and Web of Science for the literature review on the MGBA in CRF.

## Abbreviations

The following abbreviations are used in this manuscript:

CRF	cancer-related fatigue
MGBA	microbiota–gut–brain axis
HPA	hypothalamic–pituitary–adrenal
5-HT	serotonin
CNS	central nervous system
ENS	enteric nervous system
SCFAs	short-chain fatty acids
LPS	lipopolysaccharide
GABA	γ-aminobutyric acid
DA	dopamine
TRP	tryptophan
KYN	kynurenine
ACh	acetylcholine
α7nAChR	α7 nicotinic acetylcholine receptor
CRH	corticotropin-releasing hormone
ACTH	adrenocorticotropic hormone
ADT	androgen-deprivation therapy
BFI	Brief Fatigue Inventory
CRP	*C*-reactive protein
EPA	Eicosapentaenoic acid
FACIT-F	Functional Assessment of Chronic Illness Therapy-Fatigue
FFQ	food-frequency questionnaire
GI	glycemic index
LBM	lean body mass
MedDiet Score	Panagiotakos Mediterranean diet score
NSCLC	non-small-cell lung cancer
ONS	oral nutritional supplement
SF-36	Short Form-36
SI	Symptom Inventory
WC	waist circumference
WHI	Women’s Health Initiative
RCT	randomized controlled trial
QoL	quality of life
CFU	colony-forming unit
HEN	home enteral nutrition
IL	interleukin
TSF	triceps skinfold thickness
AMC	arm muscle circumference
U-CARE	medicinal-mushroom blend dietary supplement
IQR	interquartile range
HEI	Healthy Eating Index
MEDAS	Mediterranean Diet Adherence Screener
DII	dietary inflammatory index
BIA	bioelectrical impedance analysis
AMED	Alternative Mediterranean Diet score
BMI	body mass index
FFQs	Food Frequency Questionnaires
DXA	Dual-energy X-ray Absorptiometry
PG-SGA	Patient-Generated Subjective Global Assessment
MST	Malnutrition Screening Tool
NRS	Nutritional Risk Screening (NRS-2002)
MCP	Microbial cell preparation
adv	advanced
AUC	area under the ROC curve
BC	breast cancer
chemo	chemotherapy
CRT	chemoradiotherapy
FDR	false discovery rate
FMT	fecal microbiota transplantation
GSEA	gene set enrichment analysis
HNC	head and neck cancer
KEGG	Kyoto Encyclopedia of Genes and Genomes
LEfSe	linear discriminant analysis effect size
NCRF	non-cancer-related fatigue
pts	patients
QIIME2	Quantitative Insights Into Microbial Ecology 2
SNS	sympathetic nervous system
